# Explainable machine learning for early predicting treatment failure risk among patients with TB-diabetes comorbidity

**DOI:** 10.1038/s41598-024-57446-8

**Published:** 2024-03-21

**Authors:** An-zhou Peng, Xiang-Hua Kong, Song-tao Liu, Hui-fen Zhang, Ling-ling Xie, Li-juan Ma, Qiu Zhang, Yong Chen

**Affiliations:** 1 Department of the Fifth Tuberculosis, Chongqing Public Health Medical Center, Chongqing, People’s Republic of China; 2 Department of Endocrinology, First Affiliated Hospital of Anhui Medical University, Hefei, 230022 Anhui People’s Republic of China; 3 Department of Geriatrics and Special Services Medicine, Xinqiao Hospital, Third Military Medical University, Chongqing, China

**Keywords:** Infectious diseases, Respiratory tract diseases

## Abstract

The present study aims to assess the treatment outcome of patients with diabetes and tuberculosis (TB-DM) at an early stage using machine learning (ML) based on electronic medical records (EMRs). A total of 429 patients were included at Chongqing Public Health Medical Center. The random-forest-based Boruta algorithm was employed to select the essential variables, and four models with a fivefold cross-validation scheme were used for modeling and model evaluation. Furthermore, we adopted SHapley additive explanations to interpret results from the tree-based model. 9 features out of 69 candidate features were chosen as predictors. Among these predictors, the type of resistance was the most important feature, followed by activated partial throm-boplastic time (APTT), thrombin time (TT), platelet distribution width (PDW), and prothrombin time (PT). All the models we established performed above an AUC 0.7 with good predictive performance. XGBoost, the optimal performing model, predicts the risk of treatment failure in the test set with an AUC 0.9281. This study suggests that machine learning approach (XGBoost) presented in this study identifies patients with TB-DM at higher risk of treatment failure at an early stage based on EMRs. The application of a convenient and economy EMRs based on machine learning provides new insight into TB-DM treatment strategies in low and middle-income countries.

## Introduction

Tuberculosis (TB) remains a global infectious disease and one of the leading causes of death worldwide. In 2020, The World Health Organization (WHO) estimated the number of people newly diagnosed with TB was 5.8 million^1^. The End TB Strategy of WHO of 2014 aims for zero mortality and morbidity from TB^2^. However, high-risk comorbidities, such as HIV, malnutrition, and dysglycemia, are preventing people from achieving this goal. A recent study has reported that persistent dysglycemia was independently associated with unfavorable treatment outcomes (adjusted odds ratio (AOR): 6.1; 95% CI: 1.9–19.6)^3^. Thus, identifying the patients with TB-DM who are more prone to unfavorable treatment failure from a large amount of miscellaneous EMRs data at an early stage is important.

Previous studies have demonstrated a relationship between diabetes mellitus (DM) and the progression of TB^3–6^. A recent systematic review from China showed that the prevalence Of DM among TB patients was 7.8% after screening 7043 articles and 43 eligible studies. The highest prevalence was in Northeast China (21.9%), followed by the East Coast (8.3%), Western China (5.9%), and Central China (5.1%)^6^. Another previous study reported that dysglycemia influences laboratory, clinical and radiographic manifestations of patients with TB, resulting in unfavorable treatment outcomes and a higher possibility of relapse and death^4^. Therefore, to improve TB-DM treatment outcomes and ease personal and societal healthcare burdens, clinicians would be better off identifying patients who are more prone to unfavorable treatment outcomes at an early stage. Then, precision treatment strategies can aid them afterward. In sum, it is necessary to establish a stable and reliable clinical prediction model to identify the high risk of treatment failure in patients with TB-DM.

In recent years, machine learning approaches have been applied to diagnosing and treating TB, providing valuable information for clinical decision-making^7–9^. ML approaches are growing fast and have been proven to predict risk factors for various diseases based on large population datasets^10^. ML algorithms can easily integrate and interpret a vast amount of heterogeneous data, which is beyond the human’s brain power. Previous ML studies, to our knowledge, have not been employed to study the treatment outcome of TB-DM. Hence, in this study, we aimed to apply supervised and unsupervised ML algorithms to a comprehensive set of clinical, demographic, laboratory, and CT (computed tomography) data to construct an interpretable and reliable predictive ML model for the treatment failure of TB among patients with TB-DM in Chongqing Public Health Medical Center (CPHM), an infectious diseases hospital in Chongqing, in the southwest of China.

## Methods

### Study design and population

**Figure 1:**
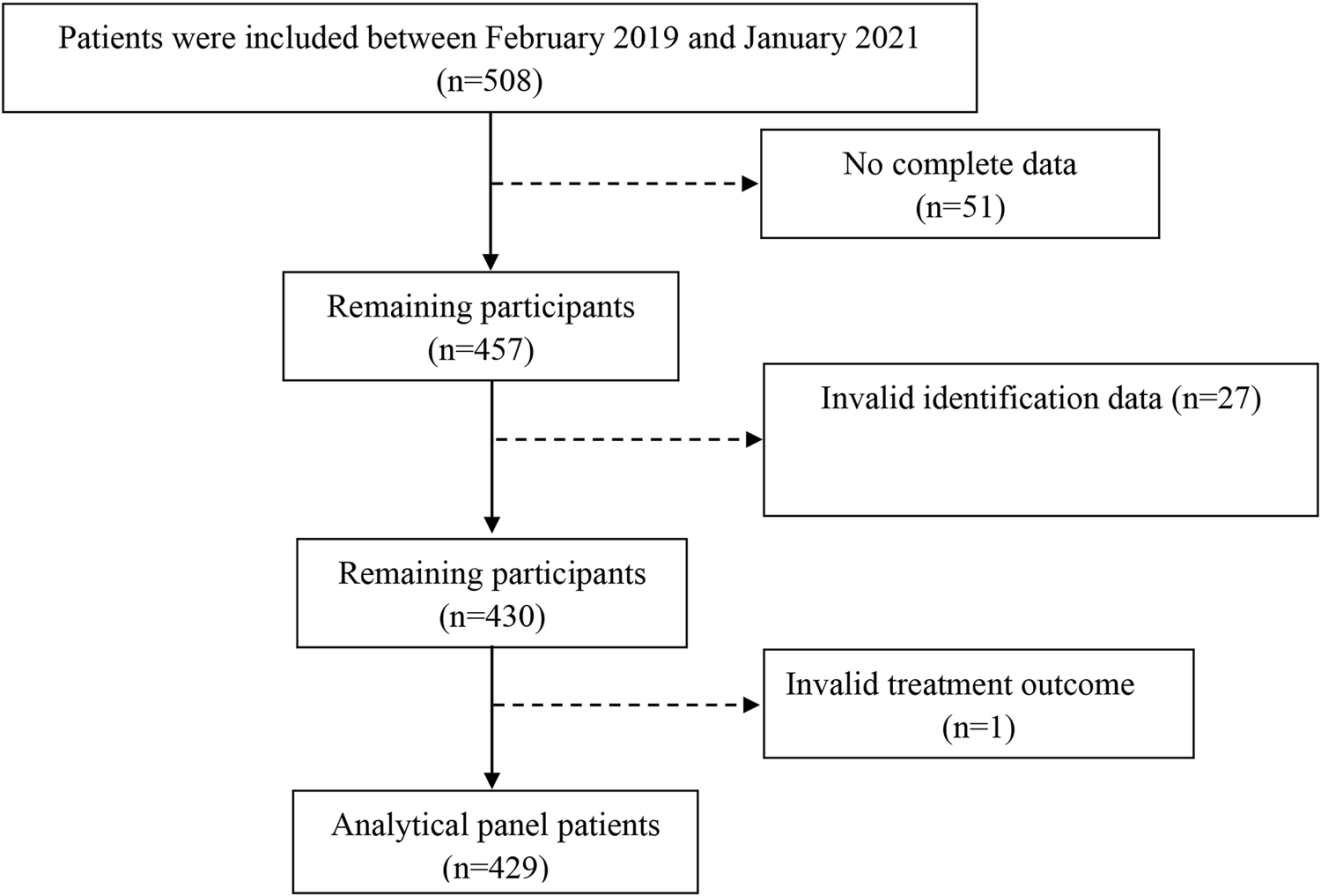
The flow chart of the study.

Five hundred and eight patients with TB-DM at CPHM between February 2019 and January 2021 were included in this retrospective study. Seventy-nine patients were excluded because of incomplete electronic medical records or lack of treatment outcome follow-up records. Finally, 429 patients were included in this study (Fig. 1). The main inclusion criteria are similar to our previous study^5^: age greater than 18 years; antituberculosis therapy for no more than one week before hospitalization in CPHM within five years; the diagnostic criterion of active PTB conforms to at least one of the following laboratory test: sputum or bronchial lavage fluid (BALF) smear positive, sputum or BALF bacterial culture positive, GeneXpert Mycobacterium tuberculosis/ rifampicin resistance in sputum or BALF positive.

### Treatment outcome

According to WHO guidelines, TB treatment outcome was defined as failure or cure^11^ (Supplementary Tables 1–2). In this study, both cured and completed treatment were identified as successful TB treatment.

### Laboratory tests

The obtained variables in this study were as follows: white blood cell count (WBC), neutrophil count (NEUT), lymphocyte count (LYMPH), monocyte count (MONO), platelet count (PLT), red blood cell count (RBC), hemoglobin (HGB), hematocrit (HCT), mean platelet volume (MPV), plateletcrit (PCT), platelet distribution width (PDW), erythrocyte sedimentation rate (ESR), C-reactive protein (CRP), total protein (TP), albumin (ALB), total cholesterol (T_CHOL), high density lipoprotein (HDL), Low Density Lipoprotein (LDL), triglyceride (TG), Alanine transaminase (ALT), Aspartate Aminotransferase (AST), total bilirubin (TBil), calcium (Ca), chlorine (Cl), kalium (K), natrium (Na), activated partial throm-boplastic time (APTT), fibrinogen (FIB), prothrombin time (PT), thrombin time (TT), urea nitrogen, creatinine, Uric Acid, fasting blood-glucose (FBG), CD4, and CD8.

### Basic feature

Age, sex, body mass index (BMI), systolic blood pressure (SBP), diastolic blood pressure (DBP), type of resistance, comorbidity ≥ 2, cough, expectoration, hemoptysis, fever, night sweats, asymptomatic, history of TB, antidiabetic (metformin, sulfonylureas, insulin), smoking, drinking history, family history of DM.

### CT feature

In this study, two experienced radiologists who were blinded to the related clinical data examined the CT images, and a senior TB expert made the final decision if the explanations of imaging results from the two radiologists were different. The number of pulmonary lobes involved, small patchy shadow, small nodules, air bronchial sign, large segmented leafy shadow, thick-walled cavity, single cavity, multiple cavities, calcification, fibrosis, lymph node enlargement, and Pleural effusion. This detail information was shown in Supplementary Table 3.

### Definition of some variables

#### Comorbidities ≥ 2

Some patients included in this study have more than 2 comorbidities, such as hypertension, dyslipidemia, pneumonia, chronic obstructive pulmonary disease, coronary heart disease, bronchiectasis, hypoproteinemia, renal failure, and so on.

#### Smoking history

Smoking status was defined as having smoked at least 100 cigarettes in life: Yes (smoker) or No (non-smoker).

#### Drinking history

It was defined as having ever consumed 1 drink of any alcoholic beverages, including liquor, beer, wine, wine coolers, and any other type of alcoholic beverage in thier entire life, not counting small tastes or sips.

#### Type of resistance

Sensitive: Drug-susceptible TB; Mono-R: mono-resistant tuberculosis; Poly-R: Poly—resistant tuberculosis; MDR: Multi-drug resistant tuberculosis; XDR: Extensively drug-resistant tuberculosis.

#### Supervised ML approach

**Figure 2:**
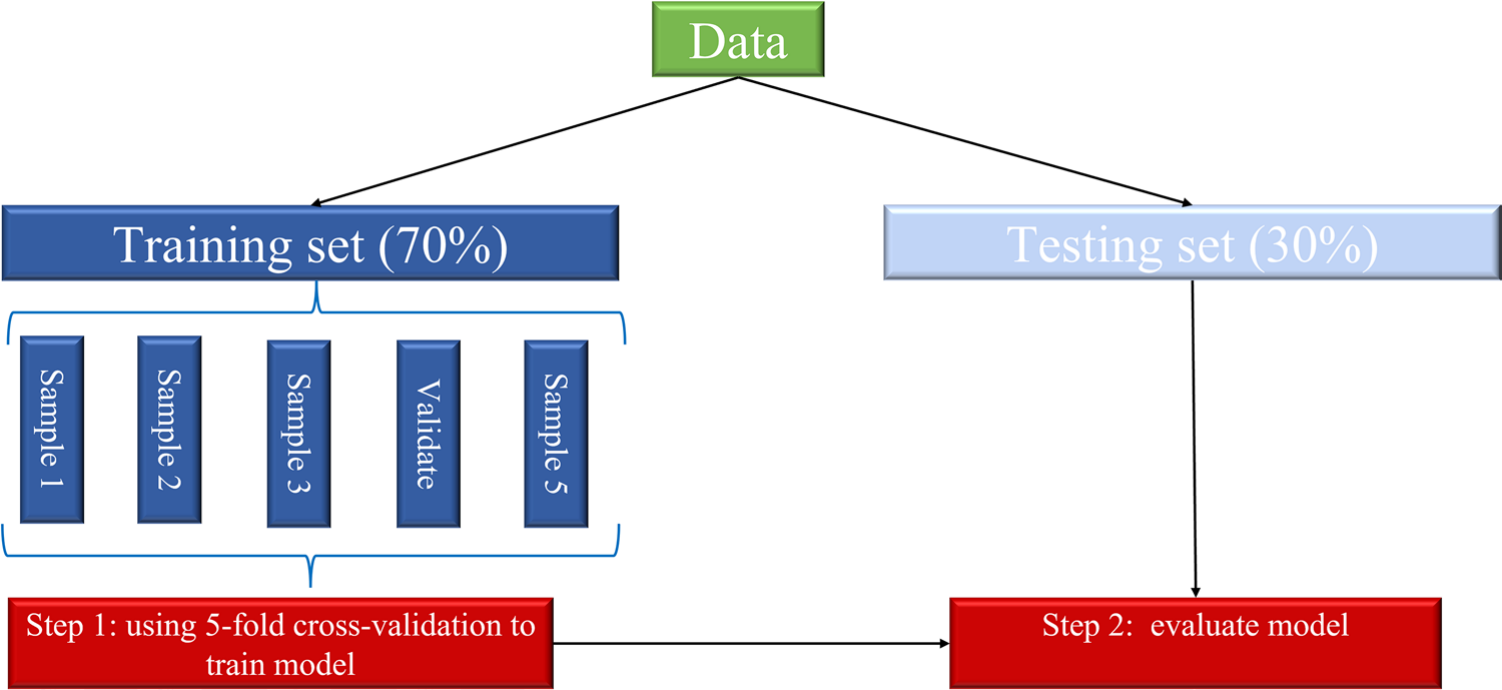
Modeling step of machine learning method (five-fold cross validation based on the data).

Given the high dimensionality of EMR data and the possible overfit, the Boruta algorithm^12^ was applied to select the best predictors of treatment failure of TB-DM in the feature selection stage. The Boruta algorism is a random forest-based feature selection method performing multiple random forest runs to compare shuffled random variables to the original variables. Then, scores standing for importance are assigned to each feature. All selected features were split into rejected, tentative, and confirmed ones according to their importance scores. In brief, confirmed features that may contribute positively to the predictive model has a performance that is better than the best random feature, indicated as ‘‘shadowMax’’. Finally, those confirmed features are considered into the ML model. Then, we split the data 70%/30% temporally and adopted a fivefold Cross-Validation on the training set to estimate the skill of the model. The remain data (test set) was used to assess the models (Fig. 2).

Four models, including XGBoost algorithm, random forest (RF), support vector machine (SVM), and logistic regression (LR), were established by ML approach using the R package ‘caret’, ‘xgboost’, and ‘e1071’. Meanwhile, model performance metrics contained accuracy score, receiver operating characteristic curve (ROC), kappa value, sensitivity, specificity, precision, recall, and F1 were also evaluated. We used a grid search to configure the best combination of hyperparameters to tune the model parameters (Supplementary Table 4).

Popular feature attribution methods may be inconsistent, which means they may reduce a feature’s assigned significance when its real impact is raised^13,14^. To address this problem, we adopted SHAP (Shapley Additive exPlanation) values based on game theory, which quantifies the contribution of each feature to the models.

Comparison of the performance of the conventional statistic, ML model using all features, and ML model plus CT features with the optimal ML model.

To validate the performance of the optimal ML model, we constructed a conventional measure, logistical regression, for comparison and the ML model using all features (69 features) from the dataset. For the conventional method, based on the previous studies^3,15–17^ and the relevance of clinical practice, we selected the sex, age, BMI, smoking, alcoholism, fasting glucose, HbA1C, type of resistance, and multiple cavities as potential confounding factors to construct a multiple analysis logistic regression model. For the ML model plus CT features, the optimal ML model combined with all CT features, including the number of pulmonary lobes involved, small patchy shadow, small nodules, air bronchial sign, large segmented leafy shadow, thick-walled cavity, single cavity, multiple cavities, calcification, fibrosis, lymph node enlargement, and Pleural effusion.

### Statistical analysis

Continuous variables were represented as mean ± standard or Median, Interquartile Range (IQR; 25–75%). Normally distributed continuous variables were compared using Student’s* t*-test, while non-normally distributed continuous variables were compared using the Mann–Whitney U test. Categorical variables were expressed as percentages (%). Comparison between groups was performed using the Χ^2^ test or Fisher exact test as appropriate. The clinical application was investigated by decision curve analysis (DCA).

RStudio (version 1.4.1717) was adopted to analyze all data in this study. For all analyses, differences with p < 0.05 were statistically significant.

### Ethics approval and consent to participate

This study was approved after agreement from the Ethics Committee of Chongqing Public Health Medical Center (no. 2021-023-02-KY). Due to the retrospective nature of the study, the Ethics Committee of Chongqing Public Health Medical Center waived the requirement for patient informed consents. The patients were anonymized and their information was nonidentifiable. In general, all data in this study was obtained in accordance with the Helsinki declaration.

## Results

### Baseline characteristics according to treatment outcome of TB-DM

A total of 429 patients were included in this study (age: 56.2 ± 11.2 (mean ± median)); male: 17.2%). Treatment failure of TB-DM occurred in around one-third of the case. The baseline characteristics are summarized in Table 1.

**Table 1:** Baseline characteristics according to treatment outcome of TB.

Characteristics	Cure	Failure	P value
	N = 294	N = 135	
Age	57.1 (10.9)	54.1 (11.6)	0.011
Female	52 (17.7%)	22 (16.3%)	0.829
BMI	21.3 [19.3–23.7]	21.7 [19.9–24.1]	0.137
Type of resistance			< 0.001
MDR	5 (1.70%)	42 (31.1%)	
Mono-R	25 (8.50%)	31 (23.0%)	
Poly-R	9 (3.06%)	14 (10.4%)	
Sensitive	253 (86.1%)	35 (25.9%)	
XDR	2 (0.68%)	13 (9.63%)	
Comorbidity ≥ 2	155 (52.7%)	61 (45.2%)	0.178
Cough	254 (86.4%)	126 (93.3%)	0.053
Expectoration	185 (62.9%)	101 (74.8%)	0.021
Hemoptysis	71 (24.1%)	31 (23.0%)	0.884
Fever	45 (15.3%)	30 (22.2%)	0.106
Night sweats	24 (8.16%)	23 (17.0%)	0.010
Asymptomatic	20 (6.80%)	3 (2.22%)	0.084
History of TB	82 (27.9%)	66 (48.9%)	< 0.001
Antidiabetic			
Metformin	112 (38.1%)	47 (34.8%)	0.585
Sulfonylureas	56 (19.0%)	20 (14.8%)	0.352
Insulin	115 (39.1%)	45 (33.3%)	0.297
Smoking history	199 (67.7%)	91 (67.4%)	1
Drinking history	163 (55.4%)	57 (42.2%)	0.015
Family history of DM	49 (16.7%)	24 (17.8%)	0.884
Laboratory tests			
Sbp	125 (17.9)	125 (17.1)	0.984
Dbp	79.1 (11.5)	79.0 (10.4)	0.955
WBC	6.59 [5.46–8.08]	6.69 [5.72–8.61]	0.338
NEUT	4.63 [3.57–6.35]	5.14 [3.76–6.59]	0.219
LY	1.18 [0.81–1.60]	1.22 [0.82–1.58]	0.763
MON	0.56 [0.43–0.74]	0.55 [0.44–0.72]	0.867
RBC	4.08 [3.77–4.54]	4.27 [3.87–4.64]	0.043
Hb	119 [107–133]	122 [110–135]	0.085
HCT	36.0 [32.2–39.6]	37.1 [34.0–41.5]	0.014
PLT	247 [195–322]	230 [182–297]	0.062
PCT	0.24 [0.20–0.31]	0.22 [0.18–0.29]	0.038
MPV	9.90 [8.70–11.0]	9.80 [8.90–11.0]	0.787
PDW	15.5 [11.7–16.2]	16.0 [15.6–16.3]	< 0.001
ESR	51.0 [28.0–84.8]	49.0 [24.0–76.0]	0.134
CRP	37.1 [8.78–76.9]	39.5 [14.1–65.0]	0.653
PT	11.7 [11.1–12.4]	12.6 [11.7–13.7]	< 0.001
TT	17.4 [16.5–18.4]	15.8 [14.8–17.3]	< 0.001
FIB	4.75 [3.66–5.95]	4.25 [3.50–5.12]	0.009
APTT	27.9 [26.3–30.9]	37.6 [30.1–42.2]	< 0.001
ALT	15.5 [11.0–27.0]	16.0 [11.0–20.5]	0.501
AST	18.0 [14.0–26.0]	18.0 [13.0–24.0]	0.467
TP	63.9 [59.3–68.0]	64.6 [61.0–70.9]	0.006
ALB	35.7 [32.5–39.3]	36.9 [34.1–40.8]	0.035
TBIL	11.4 [8.65–15.2]	10.6 [8.30–15.2]	0.322
BUN	4.89 [3.63–6.38]	4.72 [3.60–5.74]	0.202
Creatinine	54.6 [44.9–68.3]	54.9 [45.3–66.6]	0.960
Urea	294 [212–392]	284 [225–388]	0.998
TG	1.27 [0.97–1.70]	1.25 [0.98–1.71]	0.775
CHO	3.88 [3.32–4.63]	4.20 [3.63–4.85]	0.016
HDL	0.93 [0.76–1.14]	1.01 [0.80–1.33]	0.002
LDL	2.55 [2.17–3.04]	2.79 [2.38–3.31]	0.002
Na	137 [134–139]	137 [134–139]	0.717
K	4.04 [3.74–4.32]	4.09 [3.70–4.35]	0.862
Ca^2+^	2.17 [2.09–2.27]	2.20 [2.10–2.30]	0.219
Cl	101 [98.3–105]	101 [97.7–104]	0.293
GLU	8.80 [6.39–12.6]	8.95 [6.28–13.0]	0.899
HbA1c	9.10 [7.73–11.1]	9.50 [7.75–11.7]	0.403
CD4	378 [256–514]	393 [256–543]	0.719
CD8	245 [158–353]	203 [151–332]	0.278
CT features			
Number of pulmonary lobes involved			0.002
0	0 (0.00%)	1 (0.74%)	
1	10 (3.40%)	9 (6.67%)	
2	50 (17.0%)	21 (15.6%)	
3	56 (19.0%)	14 (10.4%)	
4	119 (40.5%)	43 (31.9%)	
5	59 (20.1%)	47 (34.8%)	
Small patchy shadow	223 (75.9%)	119 (88.1%)	0.005
Small nodules	204 (69.4%)	116 (85.9%)	< 0.001
Air bronchial sign	41 (13.9%)	28 (20.7%)	0.102
Large segmented leafy shadow	181 (61.6%)	80 (59.3%)	0.728
Thick-walled cavity	179 (60.9%)	90 (66.7%)	0.297
Single cavity	91 (31.0%)	50 (37.0%)	0.256
Multiple cavities	92 (31.3%)	43 (31.9%)	0.997
Calcification	19 (6.46%)	20 (14.8%)	0.009
Fibrosis	23 (7.82%)	14 (10.4%)	0.492
Lymph node enlargement	54 (18.4%)	28 (20.7%)	0.654
Pleural effusion	47 (16.0%)	18 (13.3%)	0.571

### Clustering of laboratory tests patterns between two treatment outcomes

In terms of utility and convenience, the combination of the multiple blood biomarkers may outperform single in evaluating the treatment outcome of TB-DM. Thus, we assessed the prediction of treatment outcome of the combination of different blood biomarkers by the unsupervised ML approach.

### Feature selection

**Figure 3:**
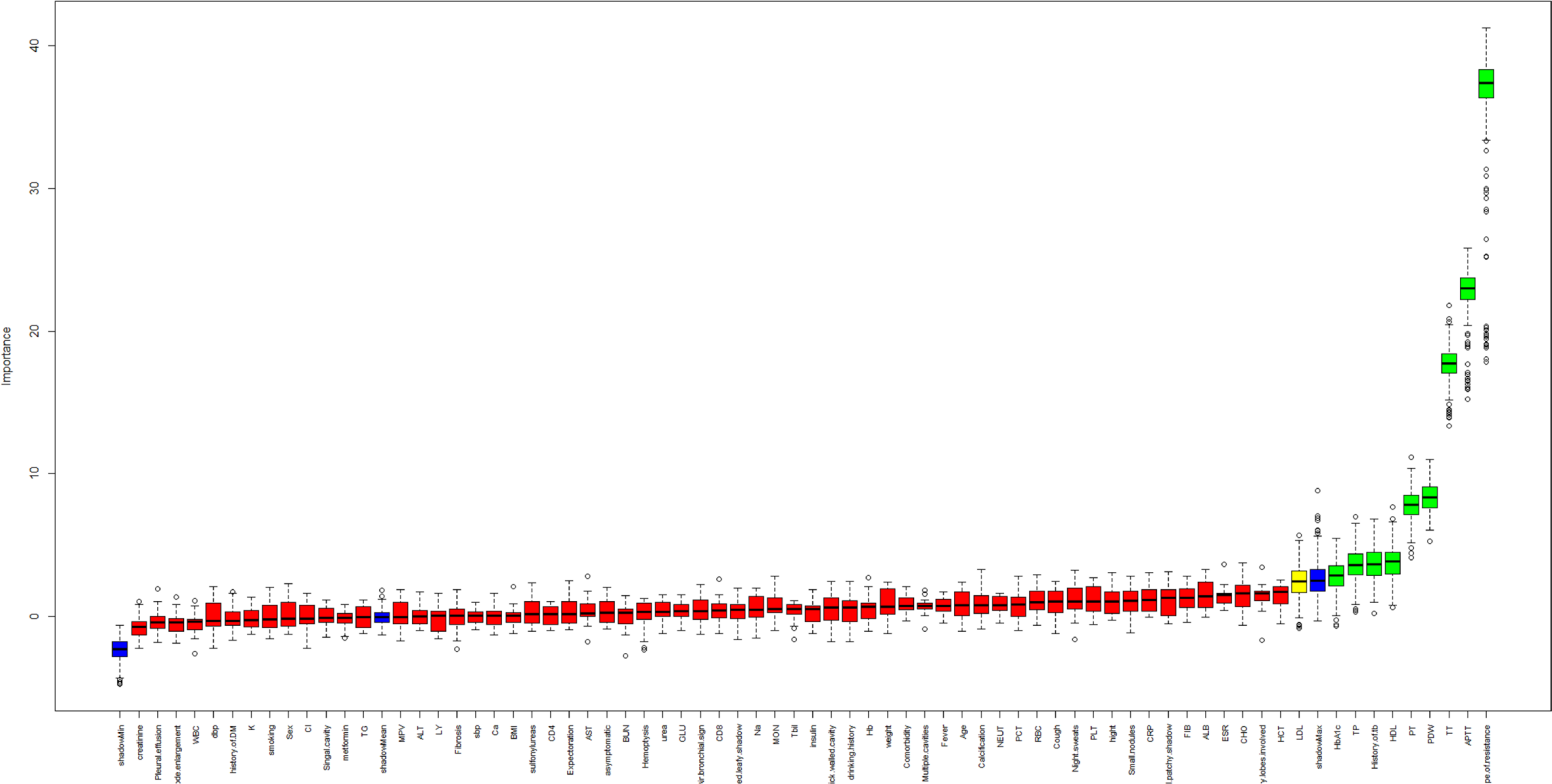
Boruta screening features results.

9 features were selected from the 69 features in this study based on the random forest-based Boruta algorithm (Fig. 3): drug-susceptible type of resistance, APTT, TT, HDL, PDW, PT, HbA1c, TP, and history of TB. In addition, other variables (rejected or tentative) with an importance score lower than shadowMax were all identified as unimportant and excluded.

### Predictive performance comparison of different classifiers

**Figure 4:**
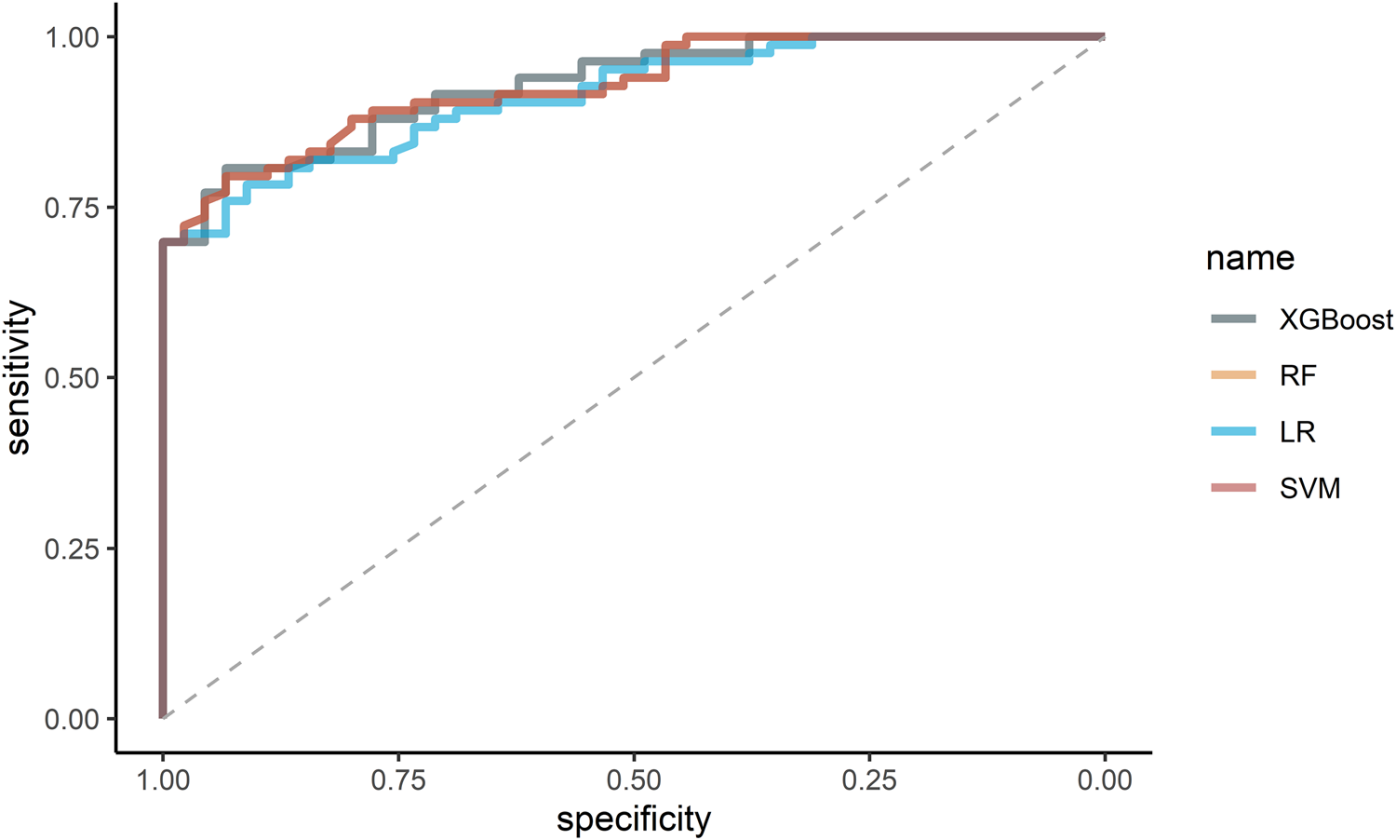
ROC curves of the four models on the testing set.

**Figure 5:**
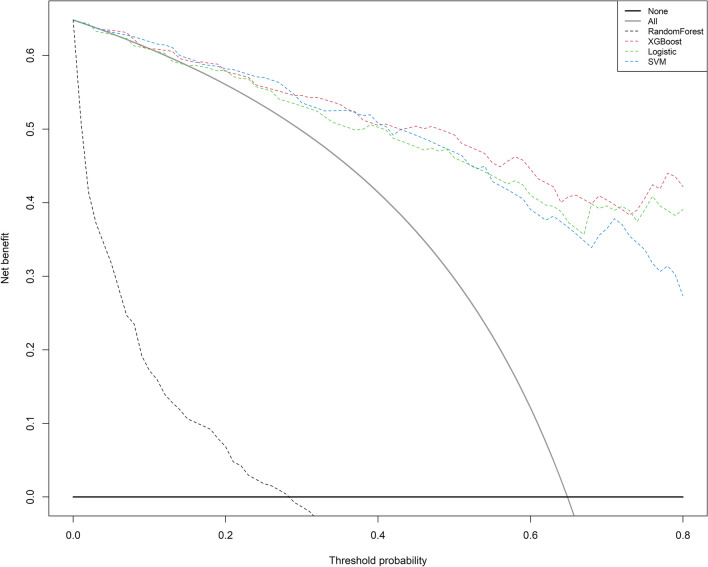
Decision curve analyses of the four models. The horizontal line here shows patients with favorable of treatment outcome, and the gray oblique line indicates patients with unfavorable of treatment outcome.

After selecting the optimal features through the Boruta algorism, we plugged them into four classifiers for further modeling, respectively. The primary confusion matrix performance and ROC scores of all ML classifiers were summarized in Table 2 and (Fig. 4). The four models have good performance as a whole (all ROC scores of models ≥ 0.7). The most promising model that predicts treatment failure of TB-DM is XGBoost, which obtained better model evaluation scores than any other ML classifiers (Table 2). Based on the decision curve analysis (DCA), the XGBoost classifier demonstrated the best net benefit along with the threshold probability than other classifiers, suggesting that XGBoost classifier was the optimal model with helpful clinical utility (Fig. 5).

**Table 2:** Model performance metrics.

Parameters	XGBoost	RF	SVM	LR
AUC	0.9281	0.9153	0.9277	0.9137
Accuracy	0.8438	0.8359	0.8047	0.8125
Kappa	0.6465	0.6308	0.5836	0.5802
Sensitivity	0.7111	0.9306	0.8000	0.6889
Specificity	0.9167	0.7111	0.8072	0.8795
Precision	0.8205	0.8523	0.6923	0.7561
Recall	0.7111	0.9306	0.8000	0.6889
F1	0.7619	0.8722	0.7423	0.7209

RF, random forest; SVM, Support Vector Machine; LR, Logistic regression; AUC, area under the curve.

The conventional method showed AUC 0.8632 and 83.7% accuracy, XGBoost using all features demonstrated 0.8858 and 0.81%, respectively. XGBoost plus.

CT features showed 0.9048 and 80.5%. While, the machine learning model, XGBoost, showed AUC 0.9281 and 84.4% accuracy. Considering the sensitivity and specificity, the conventional method showed 0.7333 and 0.8844, respectively. classifiers using all features showed 0.6222 and 0.9157, respectively. XGBoost plus CT features produced 0.6667 and 0.8795. ML model produced 0.7111 and 0.9167, respectively (Table 3).

**Table 3:** The performance metrics of the comparison between the optimal ML model, conventional method and optimal ML model using all features.

Parameters	XGBoost (Optimal ML)	Conventional method	XGBoost (69 features)	XGBoost + CT features
AUC	0.9281	0.8632	0.8858	0.9048
Accuracy	0.8438	0.8368	0.8125	0.8047
Kappa	0.6465	0.6202	0.5667	0.5604
Sensitivity	0.7111	0.7333	0.6222	0.6667
Specificity	0.9167	0.8844	0.9157	0.8795
Precision	0.8205	0.7444	0.8000	0.7500
Recall	0.7111	0.7333	0.6222	0.6667
F1	0.7619	0.7388	0.7000	0.7059

**Figure 6:**
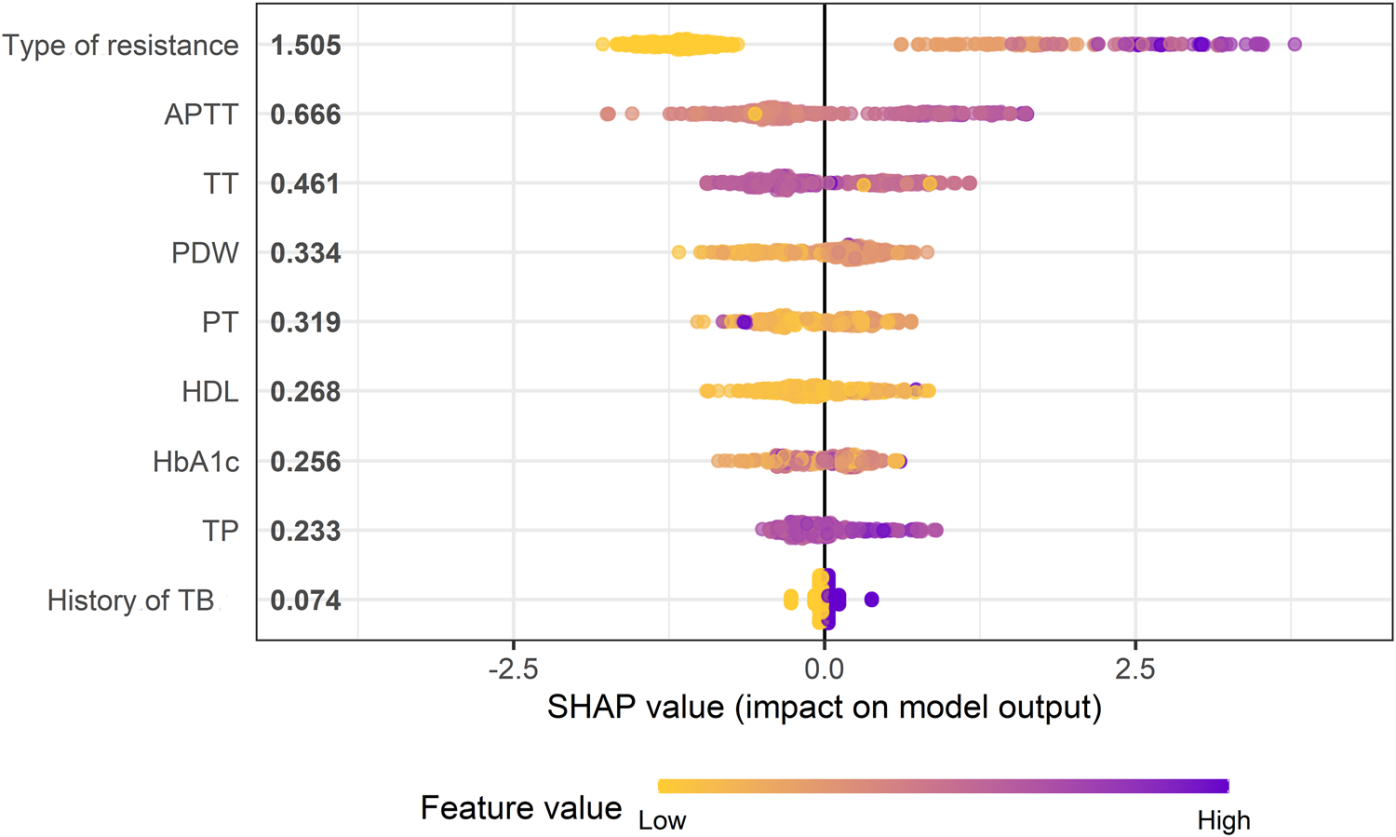
Shapley Additive exPlanations (SHAP) values for each selected feature. The higher the predictor is on the left list, the bigger the impact on model output. Each patient is represented by a dot. The x-axis represents the extent of the impact on prediction, they accumulate to represent density. The color of the dot shows the feature value (e.g., the purple color implies higher values, while yellow lower values).

After the above analysis, we calculated SHAP values of XGBoost model. Figure 6 showed the distribution of feature contributions to predictions of treatment failure of TB-DM using SHAP values of each feature for every observation. Each dot is an individual prediction. For instance, the type of resistance is associated with low and positive values on the target. Where low comes from the color and positive from the x value. In other words, people who are less drug resistant may be more likely to be cured. When APTT is high (or true) then SHAP value is high. Patients with high APTT may result in treatment failure. In addition, the high value dots of HbA1c mainly concentrates on the right side of x-axis, which means high HbA1c increases the risk of treatment failure.

## Discussion

In this study, we have shown the feasibility and stability of applying ML approaches to a comprehensive set of demographic, clinical, laboratory tests, and radiology features acquired for evaluating the treatment outcome of patients with TB-DM upon admission. Moreover, all four models we established predicted treatment failure of TB-DM with an AUC above 0.7. XGBoost is the optimal model for predicting the risk of treatment failure in TB-DM, with a high sensitivity of 71.1%, specificity of 91.7%, and an AUC of 0.9218 on the cross-validated test set. In addition, nine features were selected as predictors of treatment failure in TB-DM and certain laboratory tests were identified as critical potential predictors.

In our study, seven routine blood parameters, such as PDW, PT, TT, APTT, TP, HDL, and HbA1c, are particularly important in our models after feature selection. It is challenging to accurately interpret predictions from tree-based ML models, such as tree gradient boosting machines and random forests. Feature attribution for trees is often heuristic and not personalized for each prediction. SHAP can address the above problems. Thus, we found higher APTT, HbA1c, and PDW and lower TT, HDL and PT may increase the risk of treatment failure of TB-DM by using SHAP values to analyze the results from the XGboost model. Verma et al. have reported a significant correlation between platelet abnormalities and stroke in patients with tuberculous meningitis (TBM)^18^. In their study, they found platelet distribution width (PDW) (p < 0.001) was significantly associated with infarction in patients with TBM. In 2018, Dong et al. found hemostasis and dyslipidemia were related to exacerbated lung damage in TB, especially in patients with TB-DM, by comparing inflammatory biomarkers and hematologic and biochemical parameters between the two groups of patients, one with TB-DM and the other with TB^19^. Of note, other studies have reported the similar results^20,21^.

In our study, we demonstrated that each selected feature contributed positively or negatively to the probability of treatment failure of TB-DM, as indicated SHAP values. The resistance type is the strongest predictor of treatment outcome, and lower-level drug resistance has a more apparent negative relationship with treatment failure, as expected. Not surprisingly, patients who have a history of TB are at an increased risk of unfavorable treatment outcome. Although none of radiology features were selected into the ML models, some of their manifestations, such as multiple cavities, thick-walled cavity, the number of pulmonary lobes involved, and nodules, have been shown to be potential factors to predict the treatment outcome of TB-DM to some extent in previous studies^5,22,23^. In addition, Yang. et al. reported that radiological features, which are obtained using a single experienced radiologist reading per image, can be used for predicting drug-resistant TB (DR-TB), and that automatic discrimination between DR-TB and drug-sensitive TB (DS-TB) is possible^24^. Another study has also demonstrated that the ML model they constructed showed that radiologist observations of CT are a promising predictive method for the treatment outcome of TB^25^. Deep learning and artificial intelligence (AI) are extensively being utilized in medical image processing to assign labels and annotations to features with the aim of aiding diagnosis and prognosis. Recently, AI methods have shown superior performance compared to radiologists in distinguishing TB from non-TB using chest radiographs. However, it is important to note that radiologist evaluations of medical images are still considered the definitive benchmark for supporting the advancement of AI^26^. Clinically, distinguishing the treatment outcome of patients with TB based solely on CT images using an ML model is challenging, because CT images of TB are complicated. For instance, TB patients with different conditions exhibit multiple nodules, funicular foci, patchy dense shadows, cavities, and buds. So far, there has been no research reporting the prediction of deep learning based on CT images analysis model for the treatment outcome of pulmonary tuberculosis. Most radiomics studies based on CT with ML in TB focus on differentiating between TB and lung cancer, identifying active TB, or predicting multidrug resistance. Moreover, these studies share a common characteristic in that they typically model one feature of tuberculosis imaging, such as nodules or lung cavitation, without incorporating multiple features of tuberculosis imaging into the model. To strength our results, we applied all the full set of CT features into our optimal model, and comparison the optimal ML model and ML model plus the full set of CT features^27–31^.

Several limitations in our studies should be mentioned. Firstly, this study is a retrospective and single-center study, which is not a nationally representative. Therefore, the differences in other ethnic groups should be considered when applying our model to other populations. Secondly, there is no external validation of our models, which may restrict their applicability. Thus, further research in the future should be conducted to verify the generalizability of our findings. Thirdly, bacillary load in sputum is not routinely measured in our lab, which might influence treatment outcome. Fourth, compared to the conventional method, the ML model, XGBoost, showed the marginal improvement in AUC-ROC and lower sensitivity. The sample size in the current study was relatively small from a ML perspective, which might be partially responsible for the poor sensitivity of the prediction model.

Despite the above limitations, ML models have several advantages such as handling non-linearity and capturing complex interactions among features, which may not be effectively captured by the conventional model. The use of ML does not inherently imply automatic superiority over traditional methods, despite literature that has demonstrated so^32,33^. The effectiveness of predictive models in ML hinges on both the quality of the data utilized and the meticulous execution of the analysis. Furthermore, the results of this present study do not necessarily indicate that machine learning is completely superior to conventional statistics, but rather it highlights an inherent advantage of ML.

## Conclusions

In our study, four ML approaches for treatment failure of TB-DM yielded high predictions with functional and actionable interpretations based on ERM data. Our model is thus valuable for treating and managing TB-DM in developing countries and provides new insights for the WHO End TB Strategy.

### Supplementary Information

Supplementary Information.

## Data Availability

The datasets used and/or analyzed during the current study are not publicly available due to its proprietary nature, supporting data cannot be made openly available. But are available from the corresponding author on reasonable request.
