# Supplementary material for: Explainable machine learning for early predicting treatment failure risk among patients with TB-diabetes comorbidity
Source: Sci Rep. 2024 Mar 21;14:6814. doi: 10.1038/s41598-024-57446-8 (PMC10957874; doi:10.1038/s41598-024-57446-8)
Supplement: Supplementary file 1 — Supplementary Information. [file 41598_2024_57446_MOESM1_ESM.pdf]

Supplementary Table 1 Treatment outcomes for patients (excluding patients treated for RR-TB or MDR-TB)

Outcome	Definition
Cured	A pulmonary TB patient with bacteriologically confirmed TB at the beginning of treatment who was smear- or culture-negative in the last month of treatment and on at least one previous occasion.
Treatment completed	A TB patient who completed treatment without evidence of failure BUT with no record to show that sputum smear or culture results in the last month of treatment and on at least one previous occasion were negative, either because tests were not done or because results are unavailable.
Treatment failed	A TB patient whose sputum smear or culture is positive at month 5 or later during treatment.

Supplementary Table 2 Outcomes for RR-TB/MDR-TB/XDR-TB patients treated using second-line treatment

Outcome	Definition
Cured	Treatment completed as recommended by the national policy without evidence of failure AND three or more consecutive cultures taken at least 30 days apart are negative after the intensive phase[a].
Treatment completed	Treatment completed as recommended by the national policy without evidence of failure BUT no record that three or more consecutive cultures taken at least 30 days apart are negative after the intensive phase[a].
Treatment failed	Treatment terminated or need for permanent regimen change of at least two anti-TB drugs because of:<br>-lack of conversion[b] by the end of the intensive phase[a], or<br>-bacteriological reversion[b] in the continuation phase after conversion[b] to negative, or<br>-evidence of additional acquired resistance to fluoroquinolones or second-line injectable drugs, or<br>-adverse drug reactions (ADRs).

[a] For Treatment failed, lack of conversion by the end of the intensive phase implies that the patient does not convert within the maximum duration of intensive phase applied by the programme. If no maximum duration is defined, an 8-month cut-off is proposed. For regimens without a clear distinction between intensive and continuation phases, a cut-off 8 months after the start of treatment is suggested to determine when the criteria for Cured, Treatment completed and Treatment failed start to apply.

[b] The terms "conversion" and "reversion" of culture as used here are defined as follows: Conversion (to negative): culture is considered to have converted to negative when two consecutive cultures, taken at least 30 days apart, are found to be negative. In such a

case, the specimen collection date of the first negative culture is used as the date of conversion.

Reversion (to positive): culture is considered to have reverted to positive when, after an initial conversion, two consecutive cultures, taken at least 30 days apart, are found to be positive. For the purpose of defining Treatment failed, reversion is considered only when it occurs in the continuation phase.

Supplementary Table 3 CT features definition

CT manifestation	Description
Small patchy shadow	The lesions in the lungs were high density and the central density was higher than the periphery, and the edges were blurred
Small nodules	The CT of the nodule appears as a shadow of 2–8mm and the edges are blurred
Air bronchial sign	The phenomenon of air-filled bronchi being made visible by the opacification of surrounding alveoli. It is almost always caused by a pathologic airspace/alveolar process, in which something other than air fills the alveoli
Large segmented leafy shadow	Pathological tissues that caused by inflammation, edema, bleeding would replace the gas in the alveolar space to produce flaky shadows. The lesion that appears as a larger segment of the lung has an increased density and uniform solid shadow
Thick-walled cavity	Cavity wall on CT is >3 mm
Single cavity	Solitary cavity on the CT imaging
Multiple cavities	The number of cavities on the CT imaging ≥2
Calcification	Deposits of calcium that appear white on CT imaging and may present as parenchymal

	granulomas, mediastinal lymph nodes, and fibronodular areas of lung involvement
Fibrosis	Reticulation, architectural distortion, and honeycombing involving the lung lobes
Lymph node enlargement	Presence of enlargement of lymph nodes in one or both hila or within the mediastinum, and the lymph node ≥10 mm in short axis diameter.
Pleural effusion	Presence of a significant amount of fluid within the pleural space.

Supplementary Table 4 Hyperparameters used in RF, SVM, and XGBoost

Models	Hyperparameters
RF[a]	mtry = 3
SVM[a]	Sigma = 0.01
	C = 1.5
LR[a]	N/A
XGBoost	eta = 0.1
	gama = 1
	verbose = 1
	min_child_weight = 1.5
	nround=500
	lambda=0.5
	subsample = 1
	alpha = 1
	colsample_bytree = 1

a. RF, random forest; SVM, support vector machine; LR logistic regression;
